# Hierarchical systems in the default mode network when reasoning about self and other mental states

**DOI:** 10.1093/scan/nsag047

**Published:** 2026-06-16

**Authors:** Isaac R Christian, Samuel A Nastase

**Affiliations:** Department of Psychology, Princeton University, Princeton, NJ 08544, United States; Department of Psychology and Center for Computational Language Sciences, University of Southern California, Los Angeles, CA 90089, United States

**Keywords:** fMRI, multi-voxel pattern analysis, theory of mind, social reasoning, default mode network, self-other, metacognition

## Abstract

Humans spend considerable time contemplating the minds of others. But this ability is not limited to external agents—we also turn the lens for reading minds inward, reflecting on our own thoughts, emotions, and sense of self. Some processes involved in reasoning about minds may rely on shared mechanisms, while others may be specific to the agent under consideration. We developed a paradigm where participants performed either a mental state inference task or a control task targeting either another person presented onscreen or their own mind. Using functional MRI and multi-voxel pattern analysis, we replicate a well-established self-other gradient along the medial prefrontal wall: ventral regions encoded mental state inference patterns for self, but not other, whereas more dorsal regions encoded mental state inference for both self and other, compared to control conditions. Posterior cingulate cortex, on the other hand, differentiated the target of mental state inference. Using a cross-classification analysis, we also found that patterns in the dorsomedial prefrontal cortex, ventromedial prefrontal cortex, and right temporoparietal junction were sensitive to mental state reasoning, regardless of the target agent. Our findings reveal a functional hierarchy for mental state reasoning where certain areas support agent-specific reasoning and others support more abstract, agent-general reasoning.

## Introduction

Social reasoning relies on a theory of mind—the ability to represent the internal mental states of other agents. Because these mental states cannot be directly observed, we infer their content using the evidence available. Some of this evidence comes from internal sources, such as memories or social knowledge (Frith and Frith 1999, Tamir and Thornton 2018) and some comes from real-time external cues like eye gaze (Baron-Cohen et al. 1985) and body posture (de Gelder 2006). These mental state inferences form the basis of higher-level social reasoning, which leverages a causal understanding of mental states and behavior to inform inferences in new contexts—distinguishing it from purely statistical forms of social prediction (Ho et al. 2022, Ullman and Bass 2024, Wang et al. 2025).

Just as we can reason about other individuals, so too can we reason about our own minds. Through introspection, we consider our own perceptions, emotions, and beliefs, asking questions like “How am I feeling?” or “Am I extroverted?” The ability to form a second order representation of our own mental states and internal processes is called metacognition (Fleming et al. 2012). The processes involved in representing one’s own mind and the minds of others may be quite similar (Graziano 2013, Vaccaro and Fleming 2018, Heyes et al. 2020). In fact, some theories suggest that metacognition is merely turning the lens for reading the minds of others on oneself (Carruthers 2009, Frith 2012). According to this view, similar computations such as belief inference are deployed regardless of whether the target is oneself or another person (Gallese and Goldman 1998). Other mechanisms, in contrast, may be unique to the agent under consideration. When reasoning about our own mental states, we rely on internal cues, such as interoceptive signals (e.g. heart rate, arousal)—cues that are not available when reasoning about the mental states of other agents (Seth 2013; Picard and Friston 2014). While some social reasoning processes may generalize across agent types, others may be specific to the self or to other individuals.

Social reasoning relies on a distributed network overlapping the default mode network (DMN; Andrews-Hanna et al. 2010, Mars et al. 2012). Although associated with a range of functions (Menon 2023, Yeshurun et al. 2021), the DMN appears to be particularly sensitive to social reasoning. In contrast, other forms of reasoning (e.g. analogical reasoning) tend to recruit regions in lateral prefrontal cortex (Bunge et al. 2005, Van Overwalle 2011). Primary nodes of the DMN include the dorsal medial prefrontal cortex (DMPFC), the ventral medial prefrontal cortex (VMPFC), the posterior cingulate cortex (PCC), and bilateral temporoparietal junctions (LTPJ and RTPJ).

Despite their coordinated activity, these regions make distinct contributions depending on which agent is being reasoned about. The DMPFC is sometimes implicated in reasoning that generalizes across agents (Yoshida et al. 2010, Nicolle et al. 2012, Wittmann et al. 2016). Other studies suggest it is specifically tuned for processing others (Wagner et al. 2012, Jamali et al. 2021). On this view, the DMPFC forms part of a functional gradient along the medial prefrontal wall that tracks agent type—self or other. The VMPFC responds to the self-part of this gradient, activating when individuals evaluate their own personality (Macrae et al. 2004), affective states (Gusnard et al. 2001, Suzuki and Tanaka 2021) or adopt first-person perspectives (Vogeley et al. 2004). This self-other gradient is continuous rather than discrete. Reasoning about similar others drives greater VMPFC activity, while reasoning about dissimilar others shifts activation dorsally (Aron et al. 1991, Mitchell et al. 2006, Krienen et al. 2010).

These studies use univariate approaches to measure brain response in conditions that isolate self and other processing. The present study extends this approach by asking whether the underlying neural representations are agent general or agent specific, using multivariate pattern analysis. Participants underwent functional MRI (fMRI) while reasoning about their own mental state or the mental state of another agent performing the same task, as depicted in a video stimulus. The paradigm included four conditions: two reasoning conditions, in which participants judged whether their own mind had wandered (Self Reason) or whether another agent’s mind had wandered (Other Reason), and two control conditions requiring a judgement without explicit reasoning (Self Count, Other Count).

We hypothesized that general and agent-specific mechanisms for mental state reasoning would be differentially organized across DMN regions. Specifically, we predicted greater VMPFC sensitivity to self-reasoning and greater DMPFC sensitivity to other reasoning, each relative to their respective control conditions. We also predicted that some regions would respond to social reasoning regardless of agent, while others would be selective for agent type. We used cross-classification to identify agent-general representations and standard decoding to identify regions selective for self or other.

## Materials and methods

Twenty-eight healthy human volunteers (aged 18–50, normal or corrected to normal vision, 27 right handed, 16 female) were recruited from the community and from a subject pool sponsored by Princeton University. All subjects provided consent and received either $40 or course credit for participation. All procedures were approved by the Princeton Institutional Review Board.

**Figure 1:**
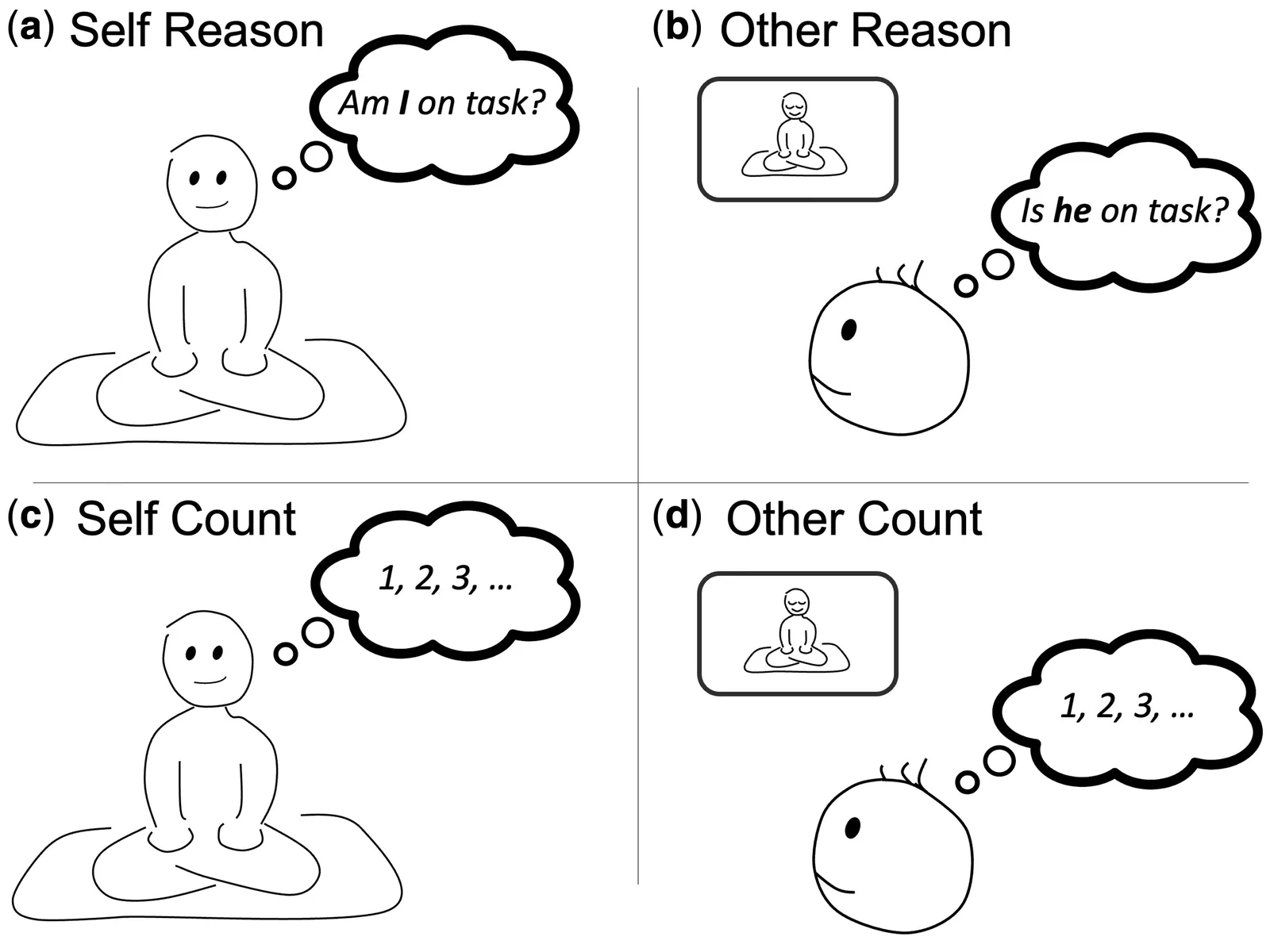
Self/other reasoning and control tasks. (a) In the Self Reason task, subjects monitored the internal content of their mind and decided when their mental state was no longer on task. (b) In the Other Reason task, subjects monitored the mental state of an agent in a video, deciding when they believed the agent was no longer on task. (c) In the Self Count task, subjects counted their own breaths and pressed a button at every fifth breath. (d) In the Other Count task, subjects counted the breaths of an agent in a video and pressed a button at every fifth breath.

The task design, shown in Fig. 1, involved four conditions: Self Reason, Self Count, Other Reason, and Other Count. In the Self Reason condition, subjects were instructed to pay attention to the rhythmic sensation of their breathing for 5 min. If at any point during this period they noticed that their mind had wandered from their breath, described as “mind wandering,” they were to indicate the moment by pressing the button and then reorient attention back to their breath and continue the task. The period right before the button press, during which participants inspected their current mental state, is of greatest interest in the present study. We hypothesized that this time period represents the most intensive mentalization about one’s own mind, and that participants would infer whether their current attention state matched a definition of “on task” at that moment. We extracted activity from this period (–3 to 0 s) before the button press. The moment of realization presumably took less than 3 s, but we used a broad 3 s window to capture variation in the mentalization process according to previous work (Hasenkamp et al. 2012).

In the Other Reason condition, participants assessed the content of another mind by watching a movie with an agent performing the Self Reason task. The film showed the agent from the waist up, revealing only the upper body and face with eyes closed. Subjects were asked to carefully attend to the agent in the movie, to try to assess on the basis of any cues (body posture, facial expression, and so on) whether the agent had suffered a moment of mind wandering, and to press the button at those moments of perceived mind wandering. We hypothesized that a mechanism involved in constructing models of someone else’s mental state should show activity in this period.

In addition to the Self Reason and Other Reason conditions, we included two control conditions. These conditions were intended to incorporate many of the same cognitive processes as the Reasoning tasks, such as attention on breath, focused control of attention, and a button press response, but they were designed to exclude the crucial moment when subjects determined whether their own mental state or the mental state of another agent was on task. For the Self Count task (Fig. 1c), subjects once again focused on their own breathing. They were instructed to count breaths, and on reaching a count of 5, to press the button and begin the count again at 1. For the Other Count condition (Fig. 1d), subjects were instructed to count the breaths of the agent in the video, and, on reaching a count of 5, to press the button and to begin the count again at 1. Piloting ensured that comparable button presses were elicited for reasoning and count conditions.

To make the stimulus videos for the Other Reason and Other Count conditions, two volunteer agents were recorded (agents A and B), for two separate 5-min films. The agents sat cross legged on a cushion and rested the right index finger on the spacebar of a computer used to record button presses. The agents were told to focus their attention on their own breathing and to press the button at the moment they realized their mind had wandered from the task. The camera was positioned to exclude view of the hands and computer (and thus of the button presses) and to capture an image of the actor above the waist.

Each subject performed 10 runs of 5 min each. First, the subject performed four runs corresponding to the four behavioral conditions in a randomized order. The same video, showing agent A, was presented for both the Other Reason and Other Count conditions. The subject then performed a second iteration of the four runs, corresponding to the four behavioral conditions in a different randomized order. For this second iteration, the video of agent B was presented for the Other Reason and Other Count conditions. In addition to the eight runs in which the behavioral task was performed, subjects performed two, 5-min runs of rest, one at the start of testing and one at the end. Between runs, participants were offered a 30-s rest that they could skip by pressing a “continue” button. The total scan time was approximately 1 h.

### fMRI acquisition and processing

Functional imaging data were collected using a 3 T MAGNETOM Skyra scanner equipped with a 64-channel head/neck coil. Gradient-echo T2*-weighted echo-planar images (EPIs) with blood oxygen level dependent (BOLD) contrast were used as an index of brain activity: TR = 1.5 s, voxel size = 2.5 × 2.5 × 3 mm. Data were preprocessed using fMRIPrep version 20.2.0. T1. Weighted volumes were skull-stripped using the OASIS template in antsBrainExtraction.sh v2.1.0. Spatial normalization through nonlinear registration to the ICBM 152 Nonlinear Asymmetrical template version 2009c was completed using the antsRegistration tool of ANTs v2.1.0 (Avants et al. 2008). Brain tissue segmentation of cerebrospinal fluid (CSF), white matter (WM), and gray matter (GM) using FAST was performed on extracted T1w images.

Functional data were slice time corrected using 3dTshift from AFNI v16.2.07 and motion corrected using MCFLIRT (FSL v5.0.9). FLIRT (FSL) performed boundary-based registration with six degrees of freedom to co-register the corresponding T1w images to functional data. Motion correcting transformations, BOLD-to-T1w transformation, and T1w-to-template warp were concatenated and applied in a single step using antsApplyTransforms (ANTs v2.1.0) with Lanczos interpolation. All functional images were low-passed (0.25) using Nilearn’s signal cleaning function. Physiological noise regressors included the first five principle components for CSF and WM for each functional run, totaling 10 aCompCor components. Head motion parameters included three translation and three rotation time series as well as censor time series for volumes with a framewise displacement (FD) exceeding 0.3 mm. For each volume with FD exceeding 0.3 mm, a vector of zeros was constructed with a value of one assigned to the time point corresponding to the offending volume. If more than 30% of volumes in a run had an average FD of 0.3 mm or larger, those runs were omitted from the analysis (9.6% of runs met this stringent criterion and were excluded). For each run, censor time series as well as 10 aCompcor components, 6 head motion parameters and 3 cosine drift parameters were inserted as regressors of no interest into the subsequent general linear models (GLMs).

A separate GLM was constructed for each button press event. To set up the GLM, we defined a period of interest as 3 to 0 s prior to the button press for each of four behavioral conditions (Self Reason, Self Count, Other Reason, Other Count). The predicted BOLD activity for each 3 s period prior to the button press was treated as a regressor of interest in a single design matrix along with the nuisance regressors. First-level regression was performed using the Nilearn’s FirstLevelModel function. Regressors of interest were convolved with a canonical hemodynamic response function (Glover 1999). Regression coefficients (beta weights) for each button press were extracted from the resulting images and used in multivariate analysis. The BOLD data were spatially smoothed using a 2 mm full width half maximum Gaussian kernel prior to regression to facilitate pattern classification across subjects.

### Multi-voxel pattern analysis

We selected five target regions of interest (ROIs) from five primary nodes of the DMN. These regions included the DMPFC, VMPFC, RTPJ, LTPJ, and the PCC. These regions were selected from a search of “mentalizing” in Neurosynth (Yarkoni et al. 2011) which yielded an average activation map across 151 studies that used the term. From this map, we created a 10 mm sphere using the maximum t-statistic as the center for each target region and used these ROI masks to extract beta weights from each subject’s whole brain image from the univariate analysis.

A linear support vector classifier (SVC) was trained on one subset of subjects and tested on a left-out subset of subjects using scikit-learn (https://scikit-learn.org/). Because some subjects pressed the button relatively few times per condition, we did not use a leave-one-subject-out procedure, which would have left too few samples for the test set in some cases. Instead, we used five-fold leave-one-group-out cross-validation across subjects. Subjects were first split into five groups (five subjects in four of the groups and eight subjects in one group). We trained the SVC on four groups and tested on one group, optimizing the parameters for the model using nested cross-validation: within each training set, we performed a grid search across the SVC regularization parameters C ranging from 10^−5^ to 10 using fivefold leave-one-group-out cross-validation and retrained the best performing model to predict the outer test set. Ultimately, this procedure resulted in five classification accuracies, which were averaged to produce the final accuracy score. To evaluate the statistical significance of that accuracy score, we created a null distribution by running 10,000 iterations of the SVC with randomly shuffled condition labels. The true decoding accuracy for each analysis was compared to the permutation-based null distribution to obtain a *P* value. We additionally defined a 95% confidence interval around the accuracy score by bootstrapping the five test statistics obtained from each fold during cross validation. These five values were resampled with replacement and their means calculated a total of 10,000 times. The upper (97.5%) and lower (2.5%) percentiles of the bootstrap means were then computed to establish confidence intervals.

This procedure was used for five analyses. Two of these analyses compared the voxel patterns of target reasoning conditions to the voxel patterns of control conditions (Self Reason compared to Self Count; Other Reason compared to Other Count). In the third test, we directly compared the voxel patterns of reasoning conditions (Self Reason compared to Other Reason). In the fourth test, we compared the voxel patterns of both control conditions (Self Count compared to Other Count).

In the final test, we trained a classifier on the distinction between the Self Reason and Self Count conditions, and then tested whether, once trained, the classifier could decode the difference between Other Reason and Other Count. This cross-classification procedure (Kaplan et al. 2015) allowed us to test whether an ROI differentiates the Reason versus Count conditions in a manner that generalizes across Self and Other conditions.

## Results

Across subjects, the mean number of button presses during a 5 min run, for each of the four tasks, was as follows: Self Reason, mean (*M*) = 10.86, SEM = 0.81; Self Count, *M* = 12.05, SEM = 0.53; Other Reason, *M* = 11.16, SEM = 1.00; and Other Count, *M* = 11.75, SEM = 0.39. Button press counts did not significantly differ between Reason and Count conditions or between Self and Other conditions (2 × 2 within-subjects ANOVA: for Reason versus Count conditions, *F* = 0.78, *P* = 0.38; for Self versus Other conditions, *F* = 0.08, *P* = 0.78; for interaction, *F* = 0.14, *P* = 0.71).

**Figure 2:**
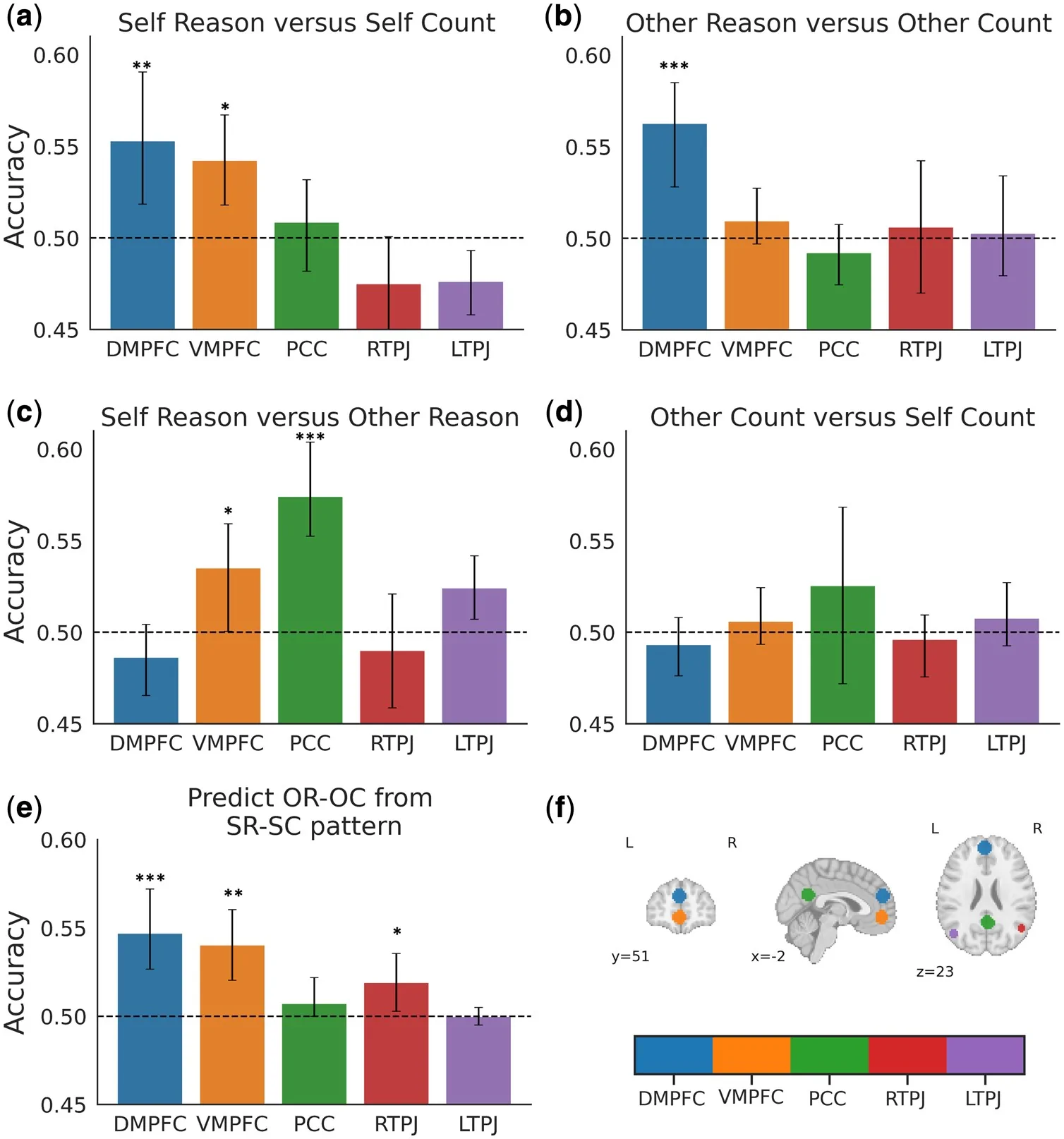
Decoding neural representations for self/other reasoning. (a) Classification results for Self Reason versus Self Count tasks across five ROIs associated with mentalizing. Activity patterns correspond to the 3 s period during which participants reasoned if they were on task or if their breath count had reached five. Dotted line shows chance accuracy of 50%. Statistical significance was assessed using a permutation test (*** *P *< 0.001, ** *P *< 0.01, * *P *< 0.05). Error bars depict 95% bootstrap confidence intervals around the mean classification accuracy. (b) Classification results for Other Reason versus Other Count tasks. (c) Classification results for Self Reason versus Other Reason tasks. (d) Classification results for Other Count versus Self Count tasks. (e) Cross-classification results when training the classifier to distinguish activity patterns for Self Reason versus Self Count tasks, and then testing the generalization of the trained classifier to the Other Reason versus Other Count tasks. (f) Five ROIs in the DMN associated with mentalizing.

The factorial design of our experiment allows us to both (i) test for distinctions between each of the four conditions and (ii) test for generalization of distinctions between levels of one factor across levels of the other. We use the word “hierarchy” to describe a two-level functional hierarchy where at the lower level of the hierarchy social reasoning is agent specific, and at the higher level of the hierarchy, social reasoning is more abstract and agent general. We evaluated these tests using decoding analyses across five ROIs in the DMN: the DMPFC, VMPFC, PCC, the right and left TPJ, (Fig. 2f).

First, we tested whether the pattern of activity within an ROI differentiated the Self Reason task from the Self Count task (Fig. 2a) and whether the pattern of activity for the Other Reason task differed from the Other Count task (Fig. 2b). In the Self Reason versus Self Count comparison, patterns within the DMPFC and VMPFC were distinct, at a peak accuracy of 55.3% (significantly above chance based on a permutation test, *P *< 0.01) for the DMPFC and 54.2% (*P *< 0.05) for the VMPFC. When classifying Other Reason versus Other Count, only the DMPFC showed significant decoding at 56.3% (*P *< 0.001).

These results show that Self and Other Reason tasks differ from control conditions. Other regions in the DMN, however, may be sensitive to the type of reasoning. To test this hypothesis, we directly compared Self Reason and Other Reason conditions. We observed significant classification accuracy in the PCC (accuracy = 57.4%, *P* < 0.001), a region of the DMN not implicated in the prior analyses (Fig. 2c).

In a control analysis, we trained a classifier to differentiate Self Count and Other Count. No DMN region encoded agent identity above chance. This suggests the decoding results above are not driven by low-level feature differences between conditions: DMN patterns distinguished self from other during mental state reasoning, but not during breath counting.

We found that DMPFC patterns differentiated Self Reason from Self Count and Other Reason from Other Count. These analyses test for agent selectivity relative to the control condition, but do not test whether the patterns generalize across agents. We used cross-classification to address this. A significant cross-decoding accuracy would indicate that DMPFC distinguishes social reasoning from counting in a similar way regardless of whether the target is self or other. As shown in Fig. 2e, the decoding is significant for the DMPFC, with a peak accuracy of 54.7% (*P *< 0.01). For completeness, we also performed cross-decoding for the other ROIs. We found that cross-classification was also significant in the VMPFC (accuracy = 54.0%, *P* < 0.001) and the RTPJ (accuracy = 51.92%, *P* < 0.05; Fig. 2e). Results were similar when we flipped the train/test splits, training on Other Reason versus Other Count and testing on Self Reason versus Self Count (Figure S1).

## Discussion

We assessed neural systems underlying reasoning about one’s own and another’s mental state. Our experimental design allowed us to test for a functional hierarchy from agent-specific to agent-general mental state reasoning. We found that patterns in the DMPFC, VMPFC, and PCC were sensitive to the type of agent involved (Fig. 2a–c), and that patterns in the DMPFC, VMPFC, and RTPJ predicted mental state inference irrespective of the agent in question (Fig. 2e). These findings reveal neural processes for social reasoning that are both general and specific to the agent under consideration (self or other), reflecting a form of hierarchical structure in the DMN where different regions engage in social reasoning at different levels of abstraction.

Social reasoning involves using a causal understanding of hidden mental states and observable behavior to make novel predictions or decisions (Ho et al. 2022, Ullman and Bass 2024, Wang et al. 2025). In the Other Reason task, participants attend to the actor on screen and use visual cues to judge if the actor’s current mental state matches the criterion for being “on task.” The self-reasoning condition parallels this process: participants attend to interoceptive cues and judge whether their own mental state is “on task.” Both tasks involve constructing a mental model of an agent’s mind and using it to make a decision.

We acknowledge that this definition is not as precise as many computational formulations (e.g. Collette et al. 2017). However, these approaches do not examine decisions about one’s own dynamic internal processes, instead defining self-oriented processing within a reinforcement learning framework as actions made for oneself (Nicolle et al. 2012) or one’s own value preferences (Garvert et al. 2015, Campbell-Meiklejohn et al. 2017), for instance. Our Self Reason task, in contrast, characterizes a reasoning process that requires explicit introspection and decisions about internal processes.

When comparing social reasoning to control conditions, we replicated a functional gradient along the medial wall of prefrontal cortex sensitive to the kind of agent—self or other—involved in social reasoning (Amodio and Frith 2006, Denny et al. 2012, Wagner et al. 2012, Bzdok et al. 2013). The VMPFC, compared to the DMPFC, showed greater decoding accuracy for the self-agent and the DMPFC, compared to the VMPFC, showed greater decoding accuracy for the other agent. This self-other structure is found in a variety of paradigms including value-based decision making (Sul et al. 2015, Ereira et al. 2018), social knowledge (Mitchell 2009, Wagner et al. 2012), and competitive gaming (Gallagher et al. 2002). Replicating this result gives us confidence that our paradigm effectively distinguished social from control processes.

Studies demonstrating a self-other gradient typically compare target conditions (self or other) to control conditions. However, evidence for a distinct self-other axis is less consistent when self and other conditions are directly compared. For example, Vanderwal et al. (2008) report no significant univariate differences between self and other conditions, while other studies find greater DMPFC activation for self compared to other (D’Argembeau et al. 2007, Benoit et al. 2010, Krienen et al. 2010)

When we directly compared social reasoning conditions, we found that patterns in the VMPFC and PCC distinguished which agent was being reasoned about. Whereas the VMPFC showed sensitivity in multiple contrasts, the PCC distinguished patterns only when Self and Other Reason conditions were directly compared. The PCC is typically implicated in self-oriented cognition (Brewer et al. 2013), attributing perspectives (Sperduti et al. 2011) and is more broadly involved in integrating past experiences, semantic knowledge, and perceptual information (Sugiura et al. 2005, Cavanna and Trimble 2006, Yeshurun et al. 2021). In our paradigm, agent-specific decoding accuracy may reflect the PCC’s role in integrating sensory input and internal experience to construct distinct mental models for self and other agents. This interpretation is supported by a control analysis in which low-level perceptual and attentional demands were matched (e.g. counting one’s own versus another’s breaths; Fig. 2d). Under these conditions, the PCC did not differentiate between agents, providing evidence that its sensitivity is specific to processes involving agent specificity during higher-order social reasoning.

Patterns in DMPFC and VMPFC, in contrast, were most consistently sensitive to social reasoning processes that were *not* agent specific (Fig. 2e; Fig. S1), even though they exhibited a nuanced pattern of decoding accuracy for individual agents, as discussed above. One explanation for this result is that both social- and meta-cognitive processes rely on shared capacity for abstraction (Vaccaro and Fleming 2018). Our task requires participants to explicitly (as opposed to implicitly) evaluate and track task performance of their own and another agent’s mental states. The observed generalizability of VMPFC and DMPFC across agents may therefore reflect a broader mechanism involved in abstracting, rather than a function exclusive to social processing, like social inference. These results suggest that the DMPFC, and to a lesser extent VMPFC, is positioned higher in a functional hierarchy where mental state reasoning is abstracted across types of agents (self or other); areas like PCC play an agent-specific role in reasoning, positioning them lower on the hierarchy.

## Conclusion

In sum, we use a novel task to characterize the components involved in agent-specific and agent-general processing. We find a representational hierarchy along a frontal–posterior axis sensitive to the reasoning process itself and regions sensitive to the target agent during reasoning. Our results extend conventional notions of hierarchical structure in higher-order cognition, which have largely been described in terms of higher-order abstraction relative to sensory processing (Margulies et al. 2016).

### Limitations

The RTPJ (in addition to the VMPFC) showed generalizability across agent type, despite exhibiting no decoding accuracy when discerning activation patterns between Self Reason and Self Count or Other Reason and Other Count. This may seem confusing—how can representations generalize for social reasoning across agents, yet fail to show significance in the more basic reasoning and counting comparisons for either agent? Although somewhat puzzling, these outcomes are not necessarily incompatible. One possible explanation is that the cross-classification analysis was better powered to detect differences in the signal compared to the standard classification analyses (e.g. due to different numbers of training and test samples).

An additional, non-mutually exclusive explanation is that features that are not agent specific may obscure distinctions in the standard classification tests; these features may be suppressed in the cross-classification task. Moreover, describing one’s own mental state and the mental state of another is a highly unconstrained process, with events occurring at different points in the task, various off-task heuristics used across participants and trial contexts, and additional cognitive processes (e.g. attention monitoring, sensory-motor) potentially contributing to observed effects. To solidify the RTPJ’s role in agent-general reasoning, future studies should utilize a larger sample size and consider within-subject classification analysis.

## Supplementary Material

nsag047_Supplementary_Data

## Data Availability

All data and code used in analyses are publicly available: https://github.com/isaacrc/social_reasoning_self_other.
