# Supplementary material for: Hierarchical systems in the default mode network when reasoning about self and other mental states
Source: Soc Cogn Affect Neurosci. 2026 Jun 16;21(1):nsag047. doi: 10.1093/scan/nsag047 (PMC13431780; doi:10.1093/scan/nsag047)
Supplement: nsag047_Supplementary_Data [file nsag047_supplementary_data.docx]

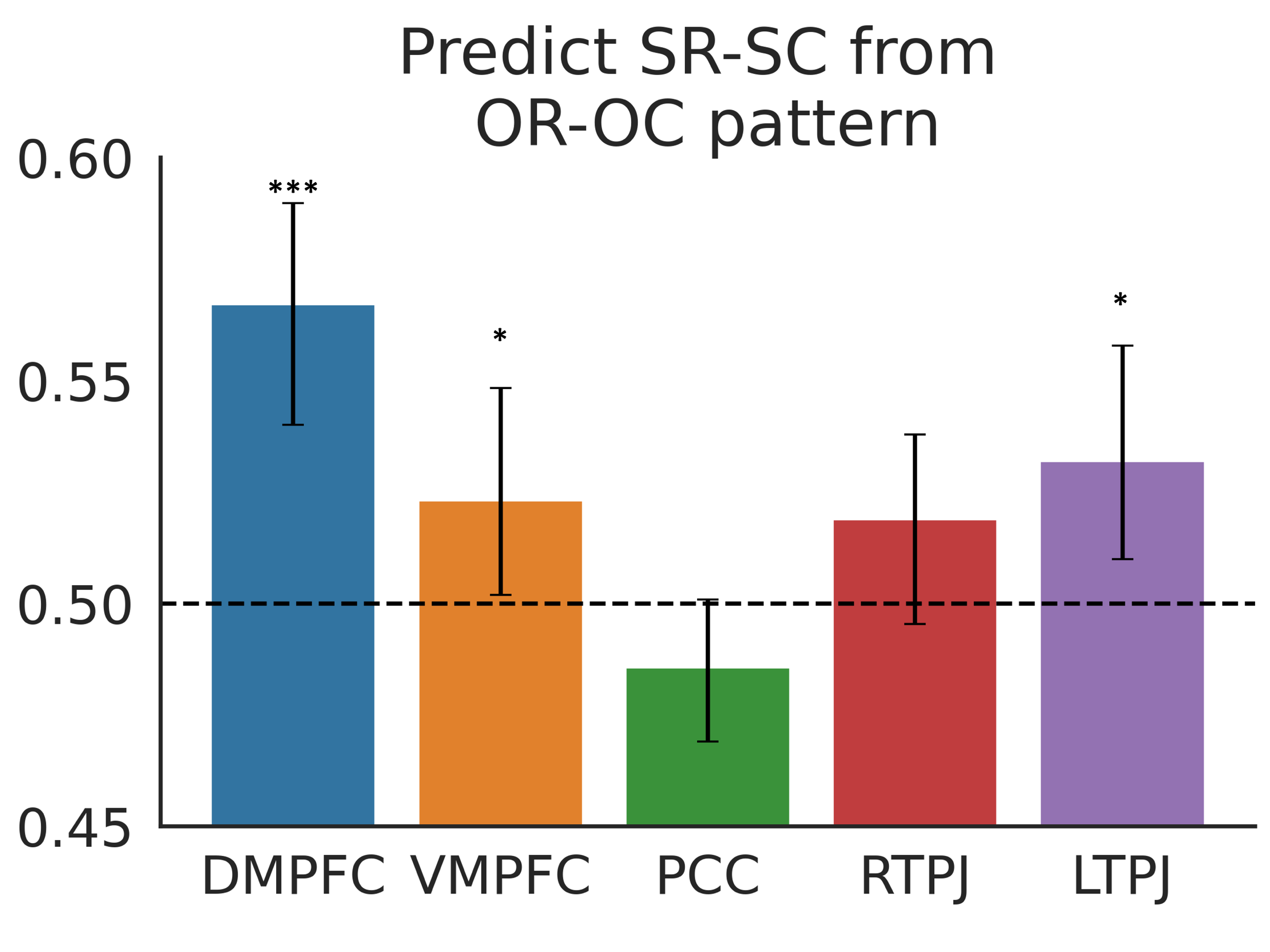


Figure 1. Cross-classification results when training the classifier to distinguish activity patterns for Other Reason versus Other Count tasks, and then testing the generalization of the trained classifier to the Self Reason versus Self Count tasks.
